# Hepatic Hydatic Cyst Causing Portal Hypertension and Cavernous Transformation in a Pediatric Patient

**DOI:** 10.14309/crj.0000000000001265

**Published:** 2024-02-07

**Authors:** Hamza Hassan Khan, Janaina Nogueira Anderson, Steven Lin, Martha Munden

**Affiliations:** 1 Division of Pediatric Gastroenterology, Hepatology, and Nutrition, Medical University of South Carolina, Charleston, SC; 2 College of Medicine, Medical University of South Carolina, Charleston, SC; 3 Department of Radiology and Radiological Sciences, Medical University of South Carolina, Charleston, SC

**Keywords:** hepatic hydatic cyst, portal hypertension, pediatrics

## CASE REPORT

**Figure 1.**
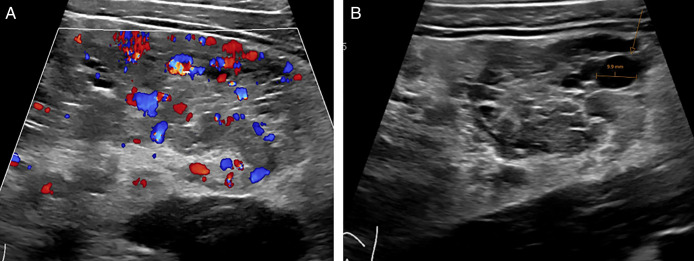
Ultrasound images with color Doppler (A) and without Doppler (B) of the marked thickened sigmoid colon, showing massively dilated veins in the bowel wall.

**Figure 2.**
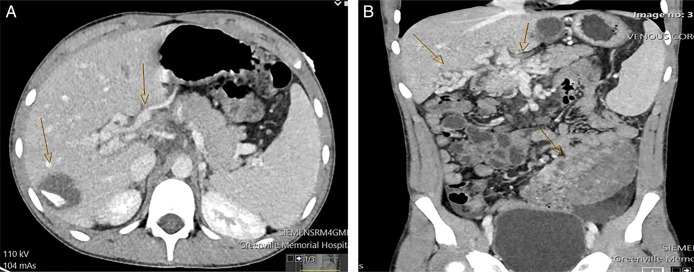
(A) Axial portal venous phase computed tomography (CT) scan showing calcification within the liver cyst right lobe (arrow) and cavernous transformation of the portal vein. (B) Portal venous phase coronal CT scan showing massive varices within the gallbladder wall and at the porta hepatis (arrows) as well as enhancement of markedly enlarged vessels within the very thickened sigmoid colon.

**Figure 3.**
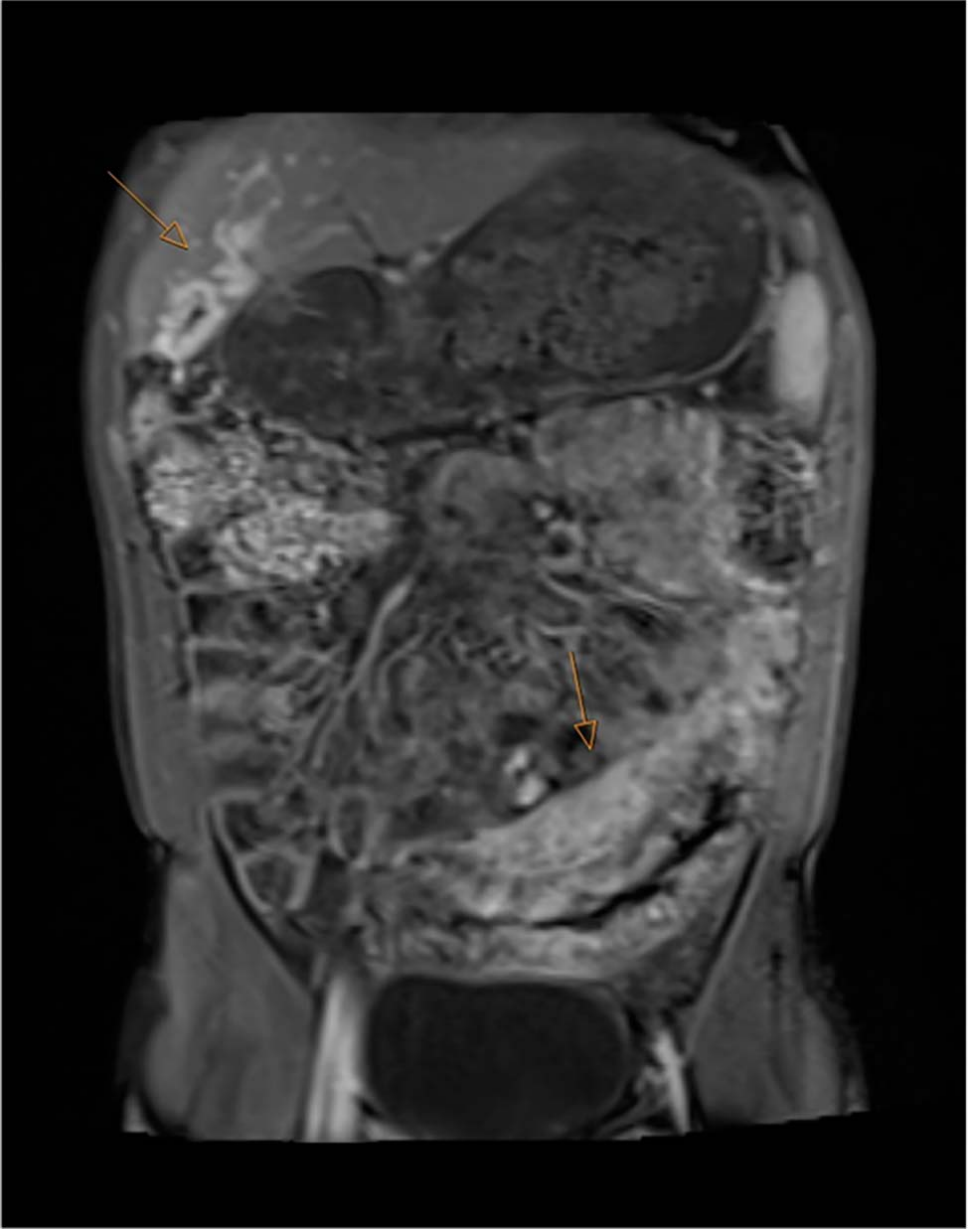
Post-contrast coronal T1 fat-saturated magnetic resonance enterography showing varices in the gallbladder wall (arrow) and massive enhancing veins within the markedly thickened sigmoid colon.

**Figure 4.**
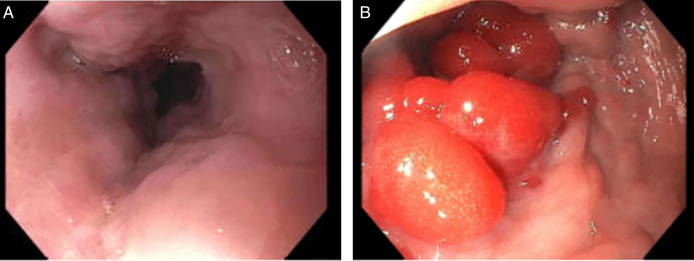
(A) Grade 3 esophageal varices in the middle esophagus. (B) Sigmoidal varices.

A 12-year-old boy with a medical history of constipation presented with 6 months’ history of hematochezia and 3 weeks’ history of headaches, diarrhea, fatigue, dizziness, and unsteady gait. Initial workup was significant for pancytopenia (white blood cell count: 3.4 K/cumm, hemoglobin: 5.9 gm/dL, platelet count: 117 K/cumm) with elevated inflammatory marker (erythrocyte sedimentation rate: 51 mm/h) and normal coagulation profile. Head computed tomography scan was normal. Abdominal ultrasound and computed tomography scan revealed 2 cystic lesions within the liver, cavernous transformation of the portal vein with massive varices surrounding the liver and involving the gallbladder wall, mild splenomegaly, and severe thickening with hyperemia and dilated vessels in the sigmoid colon (Figures 1 and 2). Abdominal magnetic resonance enterography revealed extensive varices involving the gallbladder wall, pancreatic head with cavernous transformation of the main portal vein, lesser gastric curvature, lower esophagus, sigmoid colon, and perirectal region (Figure 3). Esophagogastroduodenoscopy revealed grade 3 esophageal varices, and flexible sigmoidoscopy revealed sigmoidal varices (Figure 4). Liver biopsy revealed benign hepatic parenchyma with minimal lobular neutrophilic inflammation without fibrosis. Echinococcus serologies came back positive, and he was started on albendazole. Subsequently, he underwent splenorenal shunt with splenectomy, distal pancreatectomy, and segmental resection of the left colon with reanastomosis.

## DISCLOSURES

Author contributions: HH Khan, JN Anderson, S. Lin, and M. Munden drafted and edited the manuscript. M. Munden provided the images. All authors approved the final version. JN Anderson is the article guarantor.

Financial disclosure: None to report.

Informed consent was obtained for this manuscript.

